# Withdrawal from Dialysis and Palliative Care for Severely Ill Dialysis Patients in terms of Patient-Centered Medicine

**DOI:** 10.1155/2013/761691

**Published:** 2013-12-04

**Authors:** Hideaki Ishikawa, Nao Ogihara, Saori Tsukushi, Junichi Sakamoto

**Affiliations:** ^1^Department of Nephrology and Palliative Care, Tokai Central Hospital, Japan Mutual Aid Association of Public School Teachers, 4-6-2 Sohara-Higashijima-cho, Kakamigahara, Gifu 504-8601, Japan; ^2^Tokai Central Hospital, 4-6-2 Sohara-Higashijima-cho, Kakamigahara 504-8601, Japan

## Abstract

We treated a dementia patient with end stage chronic kidney disease (CKD). The patient also had severe chronic heart disease and suffered from untreatable respiratory distress during the clinical course of his illness. We therefore initiated peritoneal dialysis therapy (PD) as renal replacement therapy, although we had difficulties continuing stable PD for many reasons, including a burden on caregivers and complications associated with PD therapy itself. Under these circumstances we considered that palliative care prior to intensive care may have been an optional treatment. This was a distressing decision regarding end-of-life care for this patient. We were unable to confirm the patient's preference for end-of-life care due to his dementia. Following sufficiently informed consent the patient's family accepted withdrawal from dialysis (WD). We simultaneously initiated nonabandonment and continuation of careful follow-up including palliative care. We concluded that the end-of-life care we provided would contribute to a peaceful and dignified death of the patient. Although intensive care based on assessment of disease is important, there is a limitation to care, and therefore we consider that WD and palliative care are acceptable options for care of our patients in the terminal phase of their lives.

## 1. Introduction

Recently, the number of elderly dialysis patients has been increasing in Japan. According to the statistical data, nearly 60% of maintenance dialysis patients are currently over 65 years old [1].

A survey has reported that the annual mortality rate of patients on dialysis in Japan is approximately 10% [2]. In addition, the majority of dialysis patients die in a hospital [3]. Current end-of-life care for these patients has not been sufficiently discussed despite their high mortality rate [4]. The aim of this report was to explore methods for better management of end-of life care including WD and palliative care for dialysis patients with comorbid conditions and dominant illnesses admitted to a municipal hospital. Furthermore, the report has the objective of encouraging nephrologists to develop an interest in the better management of end-of-life care for maintenance dialysis patients.

Withdrawal from dialysis (WD) in end-of-life care for patients has been reported in several studies [5–9]. The benefit of dialysis is that it is a life-sustaining therapy; however it may sometimes be burdensome for patients in the terminal phase. Palliative care is also worth considering simultaneously with WD [10, 11].

Immediately after WD, due to accumulation of toxic metabolites, electrolyte imbalance, and inadequate fluid control, patients are considered to be suffering from unavoidable respiratory distress or severe pain. Hence, we need to relieve these symptoms with the help of palliative care.

While surveys of nephrologists' attitudes towards clinical practice for end-of-life care have been reported in other countries [12], there is still no comprehensive study on this issue in Japan. Dialysis patients rarely argue about WD with their nephrologist or family, and therefore there is limited discussion on improving end-of-life care of dialysis patients in Japan [13].

In our opinion, it is important for nephrologists to have considerable interest in decision making about the end-of-life care of dialysis patients.

## 2. Case Report

An 85-year-old man with severe heart failure due to dystelectasis was followed up and cared for by a cardiologist. The patient also had advanced chronic kidney disease (CKD) secondary to hypertension. The patient was admitted to hospital due to severe dyspnea. Initially, the cardiologist administered diuretic therapy; however, this treatment was not effective because of CKD. The cardiologist then consulted us as nephrologists and dialysis nurses to inquire whether dialysis was a better treatment for controlling CKD. The patient was provisionally diagnosed with advanced dementia, and we therefore concluded it was too difficult to initiate hemodialysis (HD) due to the patient being unable to keep still during therapy. We had frequent opportunities to obtain informed consent from his family, and as a result of these discussions it was decided that peritoneal dialysis (PD) managed mainly by the family would be a suitable therapy for the patient. The physical state of the patient was improved by PD, and he was able in be discharged from hospital. However, as expected, he had difficulty to accepting the procedure of PD, so he sometimes tried to draw or twist a PD tube. Hence, we supposed that even PD would be quite burdensome for his family as a caregiver.

After discharge, the patient was briefly readmitted suffering from a swollen scrotum, diagnosed as a communicating hydrocele from a hypogastric hernia complicated by PD. Although we recommended surgical treatment to continue PD, the family did not want further treatment due to further pain and physical burden for the patient and the risks associated with the operation. In that situation, as conservative care, we observed the hydrocele with discontinuation of PD. After one week, he had successfully recovered and he was able to stay without annoying pain. We were afraid that the of recurrence of communicating hydrocele as a result of restart of PD. Both we and his family were in the very difficult situation of the care for him. What kind of treatment should we prepare? Judging by his urinary volume, we considered that his residual renal function would be maintained for several weeks. We concluded that he had a right to live a peaceful and painless moment for the last period. We therefore proposed withdrawal from dialysis (WD) and palliative care as an optional treatment. We also assured the family of nonabandonment and continued careful follow-up. Despite this care, we could not obtain the patient's preference for end-of-life care. However the patient's family agreed with our choice of conservative care, and the patient was allocated to home care with follow-up and close contact being provided by our hospital.

Fortunately, the patient's respiratory distress caused by overhydration was controllable by prescribing diuretic drugs, and he was therefore able to live peacefully with his family for 147 days after WD. During this terminal phase, he was readmitted to hospital, and after the family's agreement on the risks of opioid therapy, he was administered morphine to reduce dyspnea. Palliativists played a major role in this treatment which lasted 7 days. The patient subsequently passed away peacefully, with dignity surrounded by his family.

## 3. Discussion

Despite high mortality rates in dialysis patients, only a small number of studies have investigated the preferences of these patients for end-of-life care. We consider that the current end-of-life clinical practice for severely ill dialysis patients does not meet the needs of both patients and their family members. In this report, we used the term “severely ill” for patients whose prognosis was considered critical, including conditions such as senile dementia, terminal phase of malignancy, uncontrollable critical infection diagnosed as septic shock, severe cerebral vascular disorders with persistent disturbance of consciousness, advanced chronic heart failure, and untreatable arrhythmia. Although the patient described in the case report was an extreme case, it is important to recognize that adequate management following discontinuation of dialysis may be worth considering for such patients.

**Figure 1:**
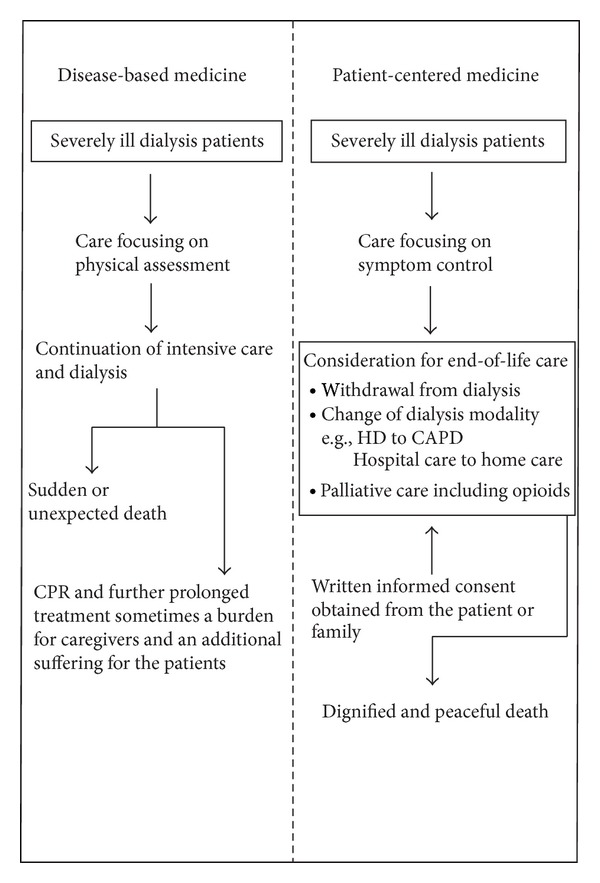
Concept of seriously ill dialysis patients showing a comparison between disease-based and patient-centered medicine. To ensure a peaceful death for these patients it is necessary to have cooperation between medical staff members such as dialysis nurses, social workers, and home doctors and nurses. Sufficient time is required to arrange the care for each patient. We consider that these clinical practices are consistent with patient-centered medicine.

Several recent studies focusing on “patient-centered medicine” have been reported [14–16], with the aim of complementing the concept of “disease-based medicine.” As shown in Ishikawa, Figure 1, we have interpreted this concept for our dialysis patients. In general, discussion of end-of-life care is regarded as an ethical issue, although attempting to assess the quality of dying so that death is dignified and peaceful is consistent with the concept of patient-centered medicine.

Although dialysis is a life-sustaining therapy, there is evidence that it does not increase life expectancy in elderly patients with severe illness [17]. When considering WD, it is also necessary to assess whether it is possible to continue dialysis by changing the modality rather than initiating WD. There are some patients in whom it is necessary to consider changing dialysis modality instead of WD. Other studies have also described the merit of PD for elderly CKD patients [18]. PD can sometimes be a feasible option for patients with severe chronic heart failure if they have difficulty continuing hemodialysis due to unstable hemodynamics or untreatable arrhythmia caused by severe cardiovascular disease. It has also been reported that PD patients have better quality of life than HD [19]. In addition to this merit, we believe that PD may be worth considering for severely ill dialysis patients as shown in our case report.

On the other hand, it is also necessary to take into account the burden of caregivers in providing PD [20, 21] as it is not possible to perform successful PD care for elderly patients without the support of caregivers, such as the patient's family members or home nurses.

Profound physiological aberrations predictably occur after WD due to accumulation of toxic metabolites, electrolyte imbalance, and inadequate fluid control. Patients may therefore experience several intolerable symptoms [22]. In our case, dyspnea was a major symptom burden that required us to institute palliative care [23, 24]. After obtaining the advice of a palliativist, we prescribed an opioid (morphine) to achieve adequate control for this symptom.

Morphine has been used widely to relieve patients of intractable pain during the terminal phase of cancer [25]. From our experience, we confirm that morphine may also be helpful against respiratory distress following WD. Despite the need for crucial management, the majority of nephrologists do not consider using opioids for dialysis patients during their end-of-life care [26].

We would also like to emphasize that nonabandonment is important in end-of-life care [15], as it is important to recognize that WD does not mean withdrawal of care. Even after WD, medical staff should maintain careful treatment to meet the needs of both patients and their families to ensure a good quality death is achieved.

However, wehave encountered some difficulties spreading the concept of patient-centered medicine. First, the majority of nephrologists in Japan are not prepared to openly discuss WD with their patients. Japanese people also may avoid discussing death or related issues and end-of-life care, which are regarded as taboo [13]. In our experience, a number of severely ill patients and their families tend to be reluctant to discuss decision making [13].

These circumstances related to the mental stress of making decisions are barriers to promoting consensus of care in both nephrologists and patients. It is therefore necessary to prepare some acceptable clinical practice guidelines on end-of-life care in Japan [27, 28].

At present, most nephrologists have not been trained to obtain the skills to undertake satisfactory management of end-of-life care [11]. We consider that this situation should be improved, which would involve education of nephrologists emphasizing the necessity of care. Experience of clinical practice for dialysis patients who are dying in hospital would also be helpful. In addition, collaboration with specialists such as a palliativist would develop a practical knowledge on the use of opioids.

In conclusion, WD and palliative care for severely ill dialysis patients are worth noting in terms of patient-centered medicine. It is hoped that clinical practice will be refined and will promote better end-of-life care for dialysis patients.

## Abbreviations

WD: Withdrawal from dialysis.
CPR: Cardiopulmonary resuscitation.

## References

[1] Shinoda T, Koda Y (2011). Clinical evaluation indices for hemodialysis/hemodiafiltration in Japan. *Contributions to Nephrology*.

[2] Nakai S, Iseki K, Itami N (2012). Overview of regular dialysis treatment in Japan (as of 31 December 2009). *Therapeutic Apheresis and Dialysis*.

[3] Yang L, Sakamoto N, Marui E (2006). A study of home deaths in Japan from 1951 to 2002. *BMC Palliative Care*.

[4] Davison SN (2010). End-of-life care preferences and needs: perceptions of patients with chronic kidney disease. *Clinical Journal of the American Society of Nephrology*.

[5] Cohen LM, McCue JD, Germain M, Kjellstrand CM (1995). Dialysis discontinuation: a “good” death?. *Archives of Internal Medicine*.

[6] Cohen LM, Germain M, Poppel DM, Woods A, Kjellstrand CM (2000). Dialysis discontinuation and palliative care. *The American Journal of Kidney Diseases*.

[7] Fissell RB, Bragg-Gresham JL, Lopes AA (2005). Factors associated with “do not resuscitate” orders and rates of withdrawal from hemodialysis in the international DOPPS. *Kidney International*.

[8] Hackett AS, Watnick SG (2007). Withdrawal from dialysis in end-stage renal disease: medical, social, and psychological issues. *Seminars in Dialysis*.

[9] Germain MJ, Davison SN, Moss AH (2011). When enough is enough: the nephrologist’s responsibility in ordering dialysis treatments. *The American Journal of Kidney Diseases*.

[10] Germain MJ, Kurella Tamura M, Davison SN (2011). Palliative care in CKD: the earlier the better. *The American Journal of Kidney Diseases*.

[11] Brown EA, Chambers EJ, Eggeling C (2008). Palliative care in nephrology. *Nephrology Dialysis Transplantation*.

[12] Holley JL, Davison SN, Moss AH (2007). Nephrologists’ changing practices in reported end-of-life decision-making. *Clinical Journal of the American Society of Nephrology*.

[13] Hirakawa Y (2011). Palliative care for elderly patients. *Nihon Ronen Igakkai Zasshi*.

[14] Bardes CL (2012). Defining ‘patient-centered medicine’. *The New England Journal of Medicine*.

[15] Quill TE, Holloway RG (2012). Evidence, preferences, recommendations: finding the right balance in patient care. *The New England Journal of Medicine*.

[16] Bowling CB, O’Hare AM (2012). Managing older adults with CKD: individualized versus disease-based approaches. *The American Journal of Kidney Diseases*.

[17] Misra M, Oreopoulos D, Vonesh E (2008). Dialysis or not? A comparative survival study of patients over 75 years of age with chronic kidney disease stage 5. *Nephrology Dialysis Transplantation*.

[18] Hiramatsu M (2003). Improving outcome in geriatric peritoneal dialysis patients. *Peritoneal Dialysis International*.

[19] Theofilou P (2011). Quality of life in patients undergoing hemodialysis or peritoneal dialysis treatment. *Journal of Clinical Medicine Research*.

[20] Riedijk SR, de Vugt ME, Duivenvoorden HJ (2006). Caregiver burden, health-related quality of life and coping in dementia caregivers: a comparison of frontotemporal dementia and Alzheimer’s disease. *Dementia and Geriatric Cognitive Disorders*.

[21] Shimoyama S, Hirakawa O, Yahiro K, Mizumachi T, Schreiner A, Kakuma T (2003). Health-related quality of life and caregiver burden among peritoneal dialysis patients and their family caregivers in Japan. *Peritoneal Dialysis International*.

[22] Chater S, Davison SN, Germain MJ, Cohen LM (2006). Withdrawal from dialysis: a palliative care perspective. *Clinical Nephrology*.

[23] Neely KJ, Roxe DM (2000). Palliative care/hospice and the withdrawal of dialysis. *Journal of Palliative Medicine*.

[24] Cohen LM, Moss AH, Weisbord SD, Germain MJ (2006). Renal palliative care. *Journal of Palliative Medicine*.

[25] Lobato RD, Madrid JL, Fatela LV (1983). Intraventricular morphine for control of pain in terminal cancer patients. *Journal of Neurosurgery*.

[26] Siegler EL, Del Monte ML, Rosati RJ, Von Gunten CF (2002). What role should the nephrologist play in the provision of palliative care?. *Journal of Palliative Medicine*.

[27] Levine DZ (2001). Shared decision-making in dialysis: the new RPA/ASN guideline on appropriate initiation and withdrawal of treatment. *The American Journal of Kidney Diseases*.

[28] Galla JH (2000). Clinical practice guideline on shared decision-making in the appropriate initiation of and withdrawal from dialysis. *Journal of the American Society of Nephrology*.

